# Changes in primary somatosensory cortex following allogeneic hand transplantation or autogenic hand replantation

**DOI:** 10.3389/fnimg.2022.919694

**Published:** 2022-10-06

**Authors:** Benjamin A. Philip, Kenneth F. Valyear, Carmen M. Cirstea, Nathan A. Baune, Christina Kaufman, Scott H. Frey

**Affiliations:** ^1^Department of Psychological Sciences, University of Missouri, Columbia, MO, United States; ^2^Program in Occupational Therapy, Washington University School of Medicine, St. Louis, MO, United States; ^3^School of Human and Behavioural Sciences, Bangor University, Bangor, United Kingdom; ^4^Department of Physical Medicine and Rehabilitation, University of Missouri, Columbia, MO, United States; ^5^Department of Cardiovascular and Thoracic Surgery, University of Louisville School of Medicine, Louisville, KY, United States

**Keywords:** amputation, somatosensory cortex, replantation, cortical organization, deafferentation, hand transplantation, tactile sensation

## Abstract

Former amputees who undergo allogeneic hand transplantation or autogenic hand replantation (jointly, “hand restoration”) present a unique opportunity to measure the range of post-deafferentation plastic changes in the nervous system, especially primary somatosensory cortex (S1). However, few such patients exist, and previous studies compared single cases to small groups of typical adults. Here, we studied 5 individuals (*n* = 8 sessions: a transplant with 2 sessions, a transplant with 3 sessions, and three replants with 1 session each). We used functional magnetic resonance imaging (fMRI) to measure S1 responsiveness to controlled pneumatic tactile stimulation delivered to each patient's left and right fingertips and lower face. These data were compared with responses acquired from typical adults (*n* = 29) and current unilateral amputees (*n* = 19). During stimulation of the affected hand, patients' affected S1 (contralateral to affected hand) responded to stimulation in a manner similar both to amputees and to typical adults. The presence of contralateral responses indicated grossly typical S1 function, but responses were universally at the low end of the range of typical variability. Patients' affected S1 showed substantial individual variability in responses to stimulation of the intact hand: while all patients fell within the range of typical adults, some patient sessions (4/8) had substantial ipsilateral responses similar to those exhibited by current amputees. Unlike hand restoration patients, current amputees exhibited substantial S1 reorganization compared to typical adults, including bilateral S1 responses to stimulation of the intact hand. In all three participant groups, we assessed tactile localization by measuring individuals' ability to identify the location of touch on the palm and fingers. Curiously, while transplant patients improved their tactile sensory localization over time, this was uncorrelated with changes in S1 responses to tactile stimuli. Overall, our results provide the first description of cortical responses to well-controlled tactile stimulation after hand restoration. Our case studies indicate that hand restoration patients show S1 function within the range of both typical adults and amputees, but with low-amplitude and individual-specific responses that indicate a wide range of potential cortical neurological changes following de-afferentation and re-afferentation.

## Introduction

The primary somatosensory cortex (S1) is organized around a somatotopic map wherein each cerebral hemisphere contains a detailed map of the contralateral body surface (Penfield and Boldrey, 1937). This cortical organization changes after amputation and similar peripheral injuries (Merzenich et al., 1984; Wall and Kaas, 1986; Kaas, 2000; Osborne et al., 2018), and the nature of these changes will define the limits of peripheral-input restoration technologies such as brain-computer interfaces (Ajiboye et al., 2017) and prostheses with sensory feedback (Li et al., 2017; Valle et al., 2018). Surgical techniques such as autogenic hand replant (i.e., reattachment after amputation) and allogeneic hand transplant (i.e., from organ donor) are two such potential methods to restore function after amputation (Kokkoli et al., 2015; Shores et al., 2015). These patients provide a unique opportunity to identify the nature, progression, and possible reorganization of cortical representations after deafferenting injury.

In the typical brain, the organization of S1 is maintained through competitive, largely inhibitory interactions between cortical areas (Kaas, 1991; Jones, 2000). After unilateral peripheral injury, deafferentation disrupts this balance; according to the classical explanation of this process, deafferentation leads to an expansion of neighboring representations into the deafferented zone (Kaas et al., 1983; Merzenich et al., 1983; Calford and Tweedale, 1990; Pons et al., 1991). For example, after hand amputation in non-human primates, contralateral S1 neurons previously devoted to the hand become responsive to cutaneous stimulation of adjacently represented areas of the hemi-face, and/or residual forelimb (Florence and Kaas, 1995; Florence et al., 1998). However, more recent research suggests that this classical explanation may be incorrect or incomplete, in that sensory (Kikkert et al., 2016) and motor (Reilly and Sirigu, 2007; Wesselink et al., 2022) representations of the absent hand may persist after amputation. Overall, while strong evidence exists for the expansion of adjacent representations at the cellular level, empirical support is lacking for these changes at the functional level in humans.

In human patients with unilateral upper extremity amputation, deafferented S1 responds to stimulation of the ipsilateral intact hand (Makin et al., 2015; Valyear et al., 2020) in addition to the contralateral phantom hand (Ding et al., 2020; Osborn et al., 2020), rather than to the cortically-adjacent lower face (which would be predicted by the classical explanation). Atypical ipsilateral responses in deafferented S1 are well-documented despite some evidence for a persistent cortical representation of the absent hand, wherein acquired amputees show a cortical representation of their intact hand, with a functional relationship between this “absent hand representation” and phantom sensation (Reilly et al., 2006; Makin and Bensmaia, 2017; Wesselink et al., 2019). However, representational persistence and S1 reorganization are not mutually exclusive because S1 could reorganize in ways that do or do not disrupt a hand representation. The atypical responses in deafferented S1 may depend on interhemispheric transfer of plasticity, as observed at the cellular level in animals. After deafferentation of a rodent forepaw, ipsilateral S1 responds to intact forepaw stimulation within 60 min (Pelled et al., 2009; Pawela et al., 2010) a process that depends on interhemispheric communication (Pelled et al., 2007). In non-human primates, after amputating the distalmost portion of a digit, receptive fields expand in the deafferented contralateral zone, becoming responsive to stimulation of the remaining phalanges, and these changes are reflected almost immediately in the S1 representation of the homotopic uninjured digit located in the opposite hemisphere (Calford and Tweedale, 1990). However, the functional significance of these changes has yet to be established.

Humans also appear to show interhemispheric transfer of plasticity, such as in patients with median nerve injury (Chemnitz et al., 2015; Nordmark and Johansson, 2020), unilateral amputation (Bogdanov et al., 2012; Valyear et al., 2020), and hand transplantation (Frey et al., 2008), whose contralesional S1 becomes more responsive to ipsilateral tactile stimulation. These changes may be associated with gray matter changes that may arise from decreased manual repertoire and increased reliance on vision for control of manual dexterity (Nordmark et al., 2018). However, most previous studies have drawn conclusions about S1 representation of the absent hand from “sensorimotor” tasks without a direct tactile stimulation component, such as phantom or attempted movement tasks (e.g., Makin et al., 2013a; Wesselink et al., 2019; Kikkert et al., 2021). As a result, the human data remain inconclusive about the nature of S1's post-deafferentation response to tactile stimulation.

Hand restoration (*via* allogeneic hand transplantation and autogenic hand replantation) is likely to have a major impact on somatosensory organization, as afferent inputs return with brain-body mappings different from the pre-injury state. Hand restoration surgery leaves patients with persistent and significant limitations in basic sensory and motor functions (Breidenbach et al., 2008; Landin et al., 2012), Evidence from the motor system suggests that hand transplant patients can achieve normal function *via* increased recruitment of compensatory cortical networks (Valyear et al., 2019), and hand replant patients show atypical interhemispheric connectivity between primary motor cortices (Eickhoff et al., 2008). Other studies in the motor system following hand restoration have indicated that motor cortices can show a pre-amputation-like state after restoration of efferent activity (Vargas et al., 2009; Sirigu et al., 2011; Madden et al., 2019). However, few studies have investigated cortical organization after hand restoration in the somatosensory system.

One landmark study demonstrated that overall cortical organization in S1 remains grossly typical after hand transplantation, with typical BOLD responses in contralateral S1 during passive tactile stimulation (Frey et al., 2008). However, this previous study compared only a single hand transplant patient against four typical adult controls, and it lacked the kind of localized, controlled stimulation that would detect expansion of cortically-adjacent or contralateral representations.

To evaluate cortical organization following hand restoration, healthy individuals alone do not provide an adequate control group. The ideal control would be within-participant longitudinal data, but this is generally impossible because of the lack of pre-injury measurements. Typical adults provide a critical first step, but it is possible for cortical organization to appear “typical-like” while remaining indistinguishable from amputees, especially given the inter-individual variability in both groups (e.g., Davis et al., 1998; Handwerker et al., 2004; Philip and Frey, 2014; Makin et al., 2015).

Here, we measured cortical organization in S1 following hand restoration (transplantation and replantation) *via* well-controlled tactile stimulation to the fingers and lower face on either side of the body during fMRI. We compared these restoration patients against two comparison groups: typical adults and unilateral upper extremity amputees, two groups that differ in the responses of affected somatosensory cortex to ipsilateral stimulation (Valyear et al., 2020). Four possible outcomes exist with respect to S1 organization in hand transplant patients. First, they could be similar to typical adults but not amputees: this would reflect either a reversal of change or that the patients did not change in the first place. Second, they could be similar to amputees but not typical adults: this would suggest that these cortical changes are epiphenomenal (since the cortex remains in an amputated-like state despite some level of sensory and motor function). Third, they could be different from either amputees or controls: this would suggest that post-restoration organization involves developing new representations unlike original representations. Fourth, they could be similar to both amputees and typical controls: interpretation is difficult, but it may reflect a still-ongoing timecourse of reorganization.

The study of hand restoration patients has the potential to address outstanding questions about somatosensory and motor reorganization, but these patients are few, with fewer than 100 worldwide (dukehealth.org, 2021). Because of our limited sample size, we used a simple confidence-interval approach with minimal mathematical assumptions to describe the organization of S1 after restoration of the once-amputated hand.

## Materials and methods

### Participants

All participants gave informed consent in accordance with the University of Missouri IRB, and the experiment was approved by the local institutional review board in compliance with the Declaration of Helsinki. Participants included five hand restoration patients: three autogenic upper limb replant patients and two allogeneic upper limb transplants. Of the two transplant patients, one participated in three sessions (labeled T1s1, T1s2, T1s3; inter-session gaps of 455 and 88 days) and the other in two sessions (T2s1, T2s2; gap 385 days). The three replant patients participated in one session each (R1, R2, and R3). Demographic details are provided in Table 1. For participants with the left side affected (1/2 transplants, 3/3 replants), all data were left-right flipped as if all patients' affected hand was their right hand (and the hemisphere contralateral to the affected limb was on the left), to allow direct comparison between groups.

**Table 1:** Demographics of hand restoration patients.

**Name**	**Aff. Side**	**Dominant Side**	**Type**	**Age**	**AAA**	**YSA**	**YSS**	**YD**
T1s1	L^*^	R	Transplant	38.6	23.1	15.5	3.2	12.3
T1s2	L^*^	R	Transplant	39.8	23.1	16.8	4.4	12.3
T1s3	L^*^	R	Transplant	40.1	23.1	17.0	4.7	12.3
T2s1	R	R	Transplant	49.2	41.1	8.1	5.5	2.6
T2s2	R	R	Transplant	50.3	41.1	9.1	6.6	2.6
R1	L	R	Replant	62.2	55.7	6.5	6.5	0
R2	L	R	Replant	59.8	59.5	0.3	0.3	0
R3	L	R	Replant	40.9	34.5	6.4	6.4	0

Aff., affected; L^*^, LH transplant, but RH also injured; AAA, age at amputation; YSA, years since amputation; YSS, years since transplant/replant surgery; YD, years deafferented (i.e., between amputation and surgery).

Two comparison groups were included. The first group was unilateral upper limb traumatic amputees [*n* = 19, 7 females; 10 below-elbow; 15 right-hand; mean age 45 ± 14 years (range 20–67); mean time since amputation 15 ± 13 years (range 2–45 years)]. The second group was typical healthy adults [*n* = 29, 11 females, mean age 44 ± 14 years (range 26–70)]. Results from these amputee and typical adult participants have been previously reported, along with detailed demographics (Valyear et al., 2020).

The hand restoration group did not differ demographically from either comparison group: not in participant age (*t*-test *p* > 0.30 vs. either group), age at amputation (*t* = 0.24, *t* = −1.2 vs. amputees), or years since amputation (*p* = 0.34, *t* = 0.97 vs. amputees).

All replants, transplants, and amputees were right-handed based on self-report of past experiences before amputation (Edinburgh score > 40). All typical adults were likewise right-hand dominant (Edinburgh score > 33) (Oldfield, 1971).

Exclusion criteria for all groups included significant psychiatric or neurological illness, and factors incompatible for MRI (e.g., implanted medical devices); for typical adults, additional exclusion criteria included currently experiencing upper limb pain, or a significant history of chronic pain.

Throughout this manuscript, primary analyses treat the hand restoration data as 8 independent sessions; while this is not statistically true, statistical in/dependence is irrelevant because of the current study's single-case design: our primary analyses never involved treating the hand restoration data as a group. Our primary fMRI analyses never indicated an effect that needed *post-hoc* analysis of potential repeated-measures effects. A more complex repeated-measures design was not justifiable with the limited data from hand restoration patients, and given our goal of simple and transparent analysis over statistical conclusions.

Only transplant patients had multiple sessions per patient, so for our replant patients and comparison groups (amputees, typical adults), there is no difference between a “session” and a “participant.”

### Behavioral measurements

#### Pain and referred sensation

Amputees completed the Neuropathic Pain Scale (Galer and Jensen, 1997) to measure average phantom limb pain (PLP) and average residual limb pain (RLP), as well as the Short-Form McGill Pain Questionnaire (Melzack, 1987) to measure current PLP. Both types of pain were evaluated by asking the patient to focus their attention on each type, respectively. No patient reported difficulty differentiating between phantom and residual limb pain. Referred sensation testing was also performed by a clinician, by dragging a 4.93 Semmes-Weinstein filament for 5 cm along the bilateral upper arms, lower arms, and face. During testing, participants closed their eyes and verbally reported the location where they felt the sensation. Similar methods have been used previously to identify the presence or absence of referred sensations (Ramachandran, 1995; Hunter et al., 2003).

#### Tactile localization (Locognosia)

All hand restoration patients, all amputees, and twenty-five (of 29) typical adults were tested for their ability to localize touch (locognosia) on the palm of their hand. Touch localization is an established method for measuring cortical reorganization after amputation and nerve injury (Hawkins, 1948; Haber, 1955), and provides an ideal test for these patients because reinnervation error is associated with difficulty localizing touch on the surface of the skin.

Tactile localization performance was tested using a method introduced by Noordenbos (1972) to measure the spatial distance between a touch's actual and perceived location. In brief, participants closed their eyes while an experimenter touched a red target mark on the participant's hand, and then the participant opened their eyes and marked the location touched using an orange pen. Participants wore red goggles throughout, which prevented them from seeing their own marks or the target marks. The variable of interest was the distance between the red mark (target) and orange mark (response), measured with 1 mm precision. One block entailed 16 locations (6 on palm, 2 on each digit); each session involved 3 blocks per hand, except for T1s1 which involved 1 block per hand. Responses that were mislocalized to a digit that was different from the one that was stimulated were removed from analysis because of the difficulty of quantifying these distances (e.g., index and middle fingertips might be 2 cm apart, but that distance does not reflect peripheral nerve organization). Mislocalized digits accounted for 13.8% of data from hand restoration patients' affected hand (29 trials total out of 210; session mean 3.6 ± 4.4, range 0–11, median 1.5).

Locognosic error of typical participants is reported for the left hand, homologous to patients' intact hand (because, as described above, patient data were left-right flipped to allow comparison between groups). Left-right flips are unlikely to affect results because locognosic testing does not show differences between the left vs. right hand in healthy adults (Moore et al., 1999; Baune et al., 2014). For amputees, the intact hand is always reported.

To test whether locognosic error in hand restoration patients would differ from either comparison group, Crawford and Howell's Modified *T*-Test was used (Crawford and Garthwaite, 2005; Crawford et al., 2011). Statistical significance was defined at α = 0.025 (0.05 / 2 to apply Bonferroni multiple-comparison correction for our 2 comparisons, vs. amputees and vs. typical adults).

Repeated-measures (between sessions, thus transplant only) effects on locognosic error were tested with one-way ANOVAs on the effect of Session (equivalent to “time” since sessions are in chronological order), separately for each transplant participant. *Post-hoc* tests were performed using Tukey's honestly significant difference.

This repeated-measures approach is mathematically different from the independent-measures approach taken elsewhere in our dataset, but as described above, our approach here is a simple and transparent analysis of a rare dataset, rather than statistical validity.

Locognosic error is interpreted as “tactile localization” (low error = high localization) so that it has the same directionality as other measures of function (high = better).

### MRI

#### Somatosensory mapping

**Figure 1:**
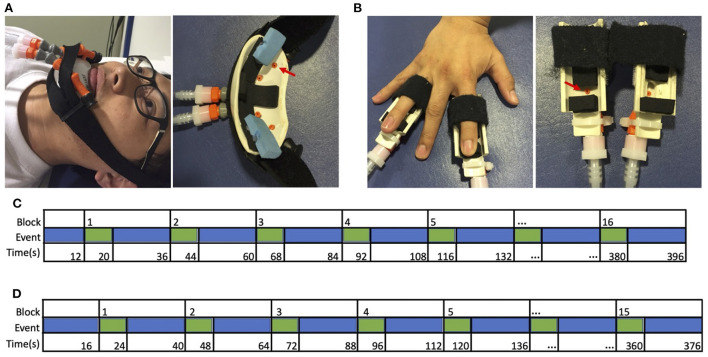
Apparatus and design. Cutaneous stimulation was applied to **(A)** the left or right lower face, or **(B)** the tips of the second and fourth digits of the left or right hand. These digits were selected to include median, radial, and ulnar nerve distributions. Sample timeline of events from a single run for **(C)** typical adults and hand restoration patients, and **(D)** amputees, from Valyear et al. (2020).

Cutaneous sensory stimulation was delivered during fMRI scanning using a custom-designed, 16-channel, computer-controlled, pneumatic apparatus (Smith et al., 2009). Four sites (three in amputees, due to absence of one hand) received puffs of compressed air: (1) Intact (left) hand index and ring fingertips, (2) Affected (right) hand index and ring fingertips, (3) intact-side (left) lower face, and (4) affected-side (right) lower face. Each site received pneumatic stimulation from two nozzles (i.e., one on each of two fingers, or two on the same side of the lower face), as illustrated in Figures 1A,B. Index and ring fingers were selected to provide stimulation to median and ulnar nerve distributions.

Stimulation was delivered in a block design. Each block comprised 8 s of stimulation (3 Hz, 20% duty cycle, 30 L/min flow rate), followed by 16s of rest. Each run comprised 15 blocks for amputees (5 blocks for each of 3 sites: intact-side face, affected-side face, and the intact hand), or 16 blocks for typical adults and hand restoration patients (4 blocks for each of 4 sites), as shown in Figures 1C,D. The sites were stimulated in a pseudorandom order within each block, counterbalanced across runs. The stimulation order was the same for each participant. Each run began with 12 s (typical adults) or 16 s (amputees) of rest. Participants completed 4 functional runs, except for one amputee who completed only 3 runs.

Throughout the experiment, participants were instructed to lay still, keep their eyes on a fixation cross, and pay attention to the air puffs. Participant's wakefulness was monitored with an eye-tracker camera (Eye-Trac 6000, Applied Science Laboratories, Bedford MA), and participant verbal self-report of wakefulness after each run. If the participant fell asleep, the run was repeated.

#### Visual mapping

To test for between-groups differences beyond the sensorimotor system, most participants (0/2 transplants, 3/3 replants, 26/29 typical adults, 10/19 amputees) performed a visual mapping task in the fMRI scanner, comprising 1 run of alternating 16 s-duration fixation and visual stimulation blocks. For details, see Valyear et al. (2020) and Supplementary material.

#### MRI parameters

Scans were performed on a Siemens (Erlangen, Germany) 3T Trio using a standard 8-channel birdcage radio-frequency coil. The session started with T1-and T2-weighted structural scans. High-resolution T1-weighted structural images were acquired using 3D MP-RAGE pulse sequence with the following parameters: TR = 2,500 ms, TE = 4.38 ms, T1 = 1,100 ms, flip angle = 8.0, 256 by 176 voxel matrix, FoV = 256 mm, 176 contiguous axial slices, thickness = 1.0 mm, and inplane resolution at 1.0 by 1.0 mm. The total durations of the T1-and T2-weighted structural scans were 8 min and 13 s, and 6 min, respectively.

Functional MRI scans were performed *via* T2^*^-weighted functional runs with echo planar imaging sensitive to the blood oxygen-level dependent contrast (BOLD-EPI) with the following parameters: TR = 3,000 ms, TE = 30 ms, flip angle = 84, 64 by 64 voxel matrix, FoV = 200 mm, 48 contiguous axial slices (no gap) with interleaved order, thickness = 3.0 mm, in-plane resolution at 4.0 4.0 mm, bandwidth = 2,004 Hz/pixel. Each BOLD scan comprised 132 volumes (396 s) for hand restoration patients and typical adults, and fewer for amputees: 125 volumes (375 s) for most participants, but due to a technical issue three amputee participants received 121 volume (363 s) runs. The first two volumes in each scan were discarded to allow steady-state magnetization to be approached. The fMRI session concluded with a double gradient echo sequence to acquire a field map used to correct for EPI distortions.

#### fMRI pre-processing

DICOM image files were converted to NIFTI format using MRIConvert software (http://lcni.uoregon.edu/~jolinda/MRIConvert/). Structural and functional fMRI data were preprocessed and analyzed using fMRIB′s Software Library(FSLv5.0, http://www.fmrib.ox.ac.uk/fsl/) (Smith et al., 2004). Non−brain structures were removed using BET. Head movement was reduced using MCFLIRT motion correction. EPI unwarping was performed to correct for distortions due to magnetic field in−homogeneities using FSL PRELUDE and FUGUE, using a separate field−map collected following the functional runs.

Functional data were spatially smoothed using a Gaussian kernel of 6 mm FWHM. Slice-time correction was applied. Intensity normalization was applied using “grand mean scaling,” wherein each volume in the dataset was scaled by the same factor to allow for valid cross-session and cross-subject statistics. High-pass temporal filtering (100 s cut-off) was applied to remove low frequency artifacts.

Functional data were registered with the high-resolution structural image using boundary-based registration (Greve and Fischl, 2009), and resampled to 1 x 1 x 1 mm resolution using FLIRT; these images were then registered to standard images (Montreal Neurological Institute MNI-152) using FNIRT non-linear registration at 12 degrees of freedom with warp resolution at 10 mm. Time series statistical analysis was carried out in FEAT v.6.00 using FILM with local autocorrelation correction (Woolrich et al., 2001).

#### fMRI data analysis

The hemodynamic response function was modeled by explanatory (predictor) variables (EVs) locked to the time course of puffer stimulation at each site: intact (left) hand, affected (right) hand, intact-side (left) face, and affected-side (right) face. For amputees, the affected (absent) hand was included in the model as an empty EV. Additional covariates of no interest were included based on the mean time series of the whole-brain, and single-point predictors for each time point of high-motion outliers. Outliers were identified within each run as time points with framewise displacement exceeding 1.5^*^interquartile range above the third quartile.

Using these EVs, first-level contrasts of parameter estimates (COPEs) were calculated for each of the following contrasts: intact hand > Rest, affected hand > Rest, intact-side (left) face > Rest, and affected-side (right) > Rest.

Second-level analyses were performed for each session by combining first-level analyses (i.e., four runs) using a fixed-effects model. Z-statistic (Gaussianized T) images were thresholded at z > 3.1, corrected for multiple comparisons using a cluster-size significance threshold of *p* < 0.05.

The top-level analysis used the second-level (subject) analyses as inputs for between-group contrasts using a mixed-effects model *via* FSL FLAME 1. Data from left-hand amputees were left-right flipped to enable combining with right-hand amputees. Three independent third-level analyses were performed: (1) average of amputee participants; (2) average of typical adult participants; (3) group-difference between amputees and typical adults. An inclusive mask was applied to these data. The mask was defined using an “OR” function to combine resultant maps specified in either group, separately, by the contrasts: Intact Hand > Rest; Affected Hand > Rest (valid only for typical adults and restoration patients, not amputees); Intact-Side Face > Rest; Affected-Side Face > Rest. This method selects voxels showing significant task-related activity in response to any stimulation condition (vs. rest), in either group. This makes subsequent tests more sensitive (in this case, specifically the contrasts between groups) by reducing the number of voxels considered for correction for multiple comparisons.

#### ROI analysis

**Figure 2:**
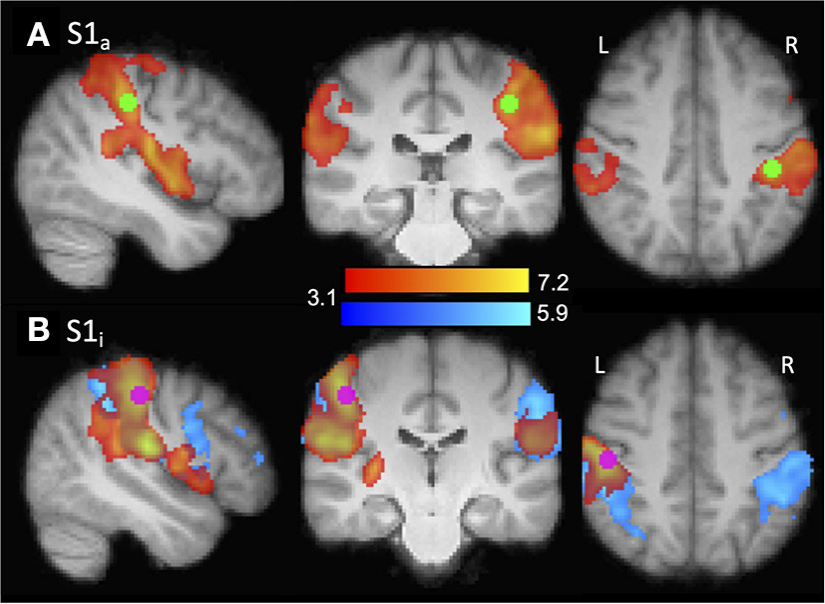
Illustration of ROI localization. ROIs (purple/green) were functionally defined from the typical adult group contrasts shown (red/yellow). **(A)** S1a. Contrast: right hand > rest. ROI center: X = −42, Y = −30, Z = 42. Amputee group contrast also shown (blue). **(B)** S1i. Contrast: left hand > rest. ROI center: X = 48, Y = −22, Z =46.

Left and right hemisphere S1 hand ROIs were functionally defined on the basis of typical adults' (group) data, hereafter referred to as the affected (S1_a_) and intact (S1_i_) sensory hand ROIs. For each ROI, the defining contrast was stimulation of the contralateral hand > rest. The voxel with the highest Z-value was identified (S1_a_: MNI coordinates X = −42, Y = −30, Z = 42; S1_i_ X = 48, Y = −22, Z = 45), a 5 mm-diameter-sphere was centered on this coordinate, and significantly active voxels (thresholded at z > 3.1) within the sphere were included if they had a ≥ 25% chance of being in the S1 complex (Brodmann's areas 1, 2, or 3) according to the Juelich Histological Atlas (Geyer et al., 2000). ROI analyses were performed at the second level (i.e., after data were transformed to MNI space). The ROIs and group data are shown in Figure 2. Left-hand amputees were retained in the ROI analysis, with their data left-right flipped, as described above.

To ensure that ROIs were never defined and evaluated using the same data, a leave-one-subject-out (LOSO) analysis (Esterman et al., 2010) was used. When a contrast would otherwise be drawn from the same condition as was used to create the ROI (i.e., for typical adults), instead an alternative ROI was defined using the LOSO approach. The LOSO approach entailed creating a ROI for each of the 29 typical adult participants based on the other 28 participants, so that the ROI data were independent from the data used to define the ROI.

The time course of changes in image intensity across all voxels of each S1 hand ROI were extracted through FSL's Featquery, and the percent BOLD signal change (%-BSC) values were calculated for each condition (i.e., COPE) for each session.

This data pipeline differs slightly from previous publication of the typical adult and amputee data (Valyear et al., 2020), due to introduction of LOSO ROIs. Previous analyses involved inverse-warping ROIs to participant brains for single-run analyses, but this process did not work with LOSO ROIs: in three participants, the inverse-warp failed, leading to an ROI outside the brain. Therefore, for the current study, it was necessary to perform the ROI analyses at the second level. These changes introduced minor differences between the current ROI results and the previous publication: Pearson correlation between the two studies' participant mean %-BSC (median of 8 ROI/condition pairs) was *r* = 0.95.

Due to the heterogeneity of hand restoration patients, single-case analytic approaches were used. First, a single-case statistical analytic method from neuropsychology (Crawford and Howell's Modified *T*-Test; Crawford and Garthwaite, 2005) was tested as a highly conservative potential way to quantify whether S1 activity differed between individual hand restoration patients and the comparison groups. We tested the method's suitability for our dataset by measuring its ability to detect individual differences where group differences were known: differences between typical adults and amputees in S1_a_ responses to intact hand stimulation (Valyear et al., 2020). Crawford and Howell's Modified *T*-test was only able to distinguish individual amputees from the typical adult group with 55% sensitivity (10/19 amputees). It should be noted that this outcome (no reliable individual-participant differences) is expected for samples from two significantly different but overlapping groups. Regardless, because of this low sensitivity, the Modified *T*-Test approach to fMRI analysis was abandoned, and is not reported further for S1 fMRI data. To minimize publication bias, it is important to report that this approach was attempted.

To provide a simple well-established method of single-case analysis, confidence intervals and ranges were used to assess whether S1 activity differed between individual hand restoration patients and the comparison groups. This simple approach to analyzing individual cases (e.g., Frey et al., 2008; Valyear et al., 2019) allows comparisons without making statistical assumptions, and allows the reader to observe individual variability and easily make judgments based on the raw data. Here, estimates of the comparison group means were calculated as 95% confidence intervals using a bootstrap method, to avoid differences due to the different sample sizes of the two comparison groups (Wood, 2004). 25,000 bootstrap samples were used *via* a bias corrected and accelerated percentile method, using the MATLAB function “bootci” (Mathworks, Natick MA).

#### Correlational analysis between behavioral/demographic variables and ROI data

Four behavioral/demographic factors were tested for correlations with %-BSC in S1_a_ and S1_i_ ROIs during affected hand stimulation in transplant/replant patients: one sensory measure (locognosic error on the restored hand) and three demographic values (years since amputation, years since surgery, and age at test). Age at amputation was not included since it was colinear with age at test (r = 0.97).

Correlations between ROI activity and demographic variables were assessed using non-parametric Kendall's τ. Statistical significance was defined at α = 0.0125 (0.05/4 factors). However, given the sample size of 8, correlation analyses should be interpreted as illustrative rather than statistically conclusive.

## Results

### Previously deafferented somatosensory cortex shows a wide range of sensitivity to stimulation of the intact hand, and is sensitive to stimulation of the affected hand

**Figure 3:**
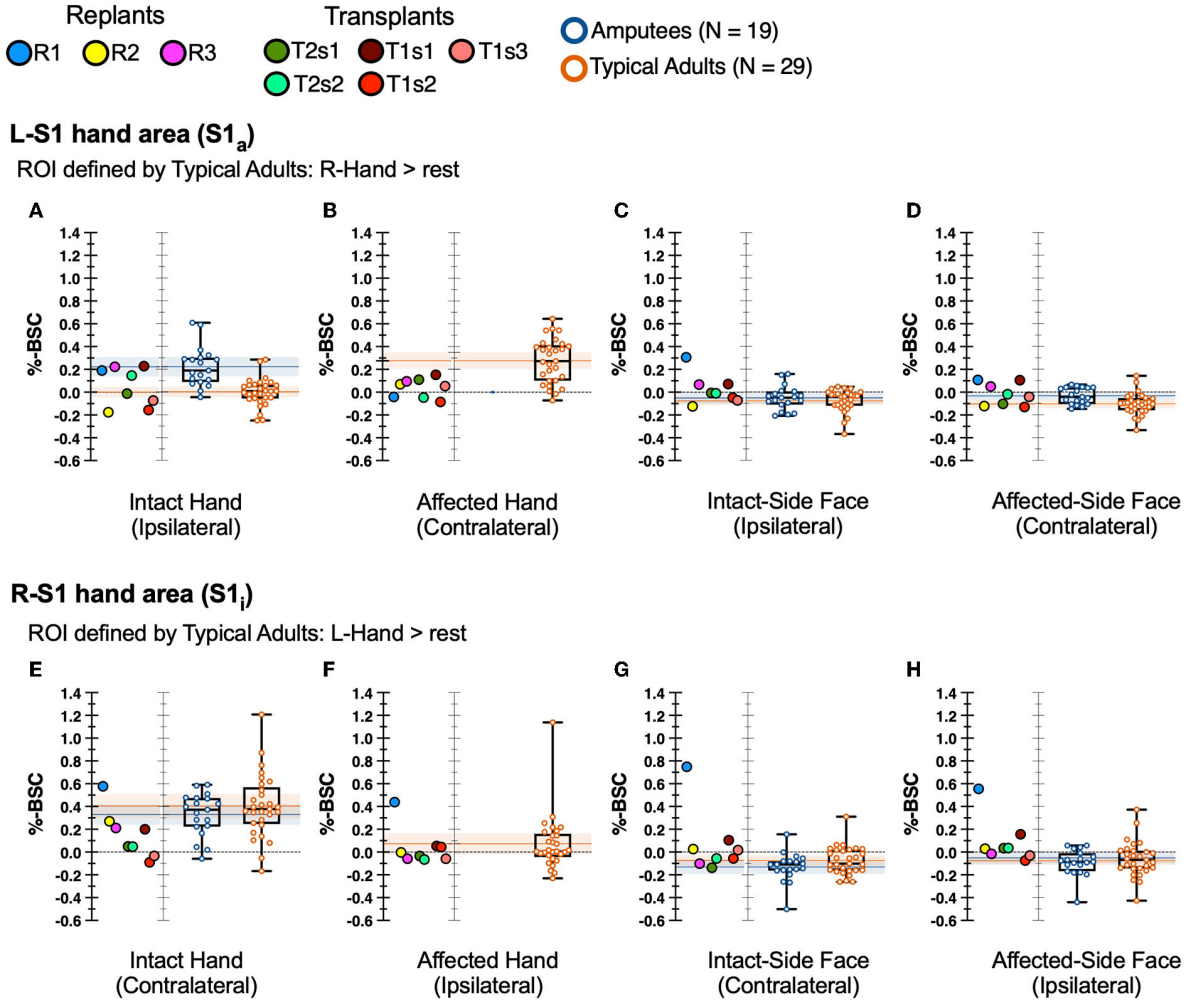
fMRI activity in hand restoration patients and comparison groups. Single points represent transplants/replants. Boxplot lines at 25^th^, 50^th^, and 75^th^ percentiles. Shaded bars: 95% confidence interval of the mean of each comparison group. Top row **(A–D)** reflects S1_a_ ROI, bottom row **(E–H)** reflects S1_i_ ROI. First column **(A,E)** represents stimulation of the intact hand. Second column **(B,F)** represents stimulation of the affected hand. Third column **(C,G)** represents stimulation of the intact-side face. Fourth column **(D,H)** represents stimulation of the affected-side face.

In the previously deafferented somatosensory cortex (S1_a_) of hand restoration patients stimulation of the intact (ipsilateral) hand produced a wide variety of responses, as shown in Figure 3A. On average, the hand restoration group (median 0.067 percent signal change, which does not change if we avoid repeated-measures effects by reducing each transplant to a single across-sessions mean) was more like typical adults (median 0.008) than amputees (median 0.189). However, at an individual-participant level, the 8 hand restoration sessions were widely distributed: 3 sessions fell within the 95% confidence interval of amputees, 1 between the confidence intervals of amputees and typical adults, 1 within the 95% confidence interval of typical adults, and 3 who were below the 95% confidence interval of controls (and amputees). Even the amputee-like participants (those with high activation magnitude) still fell within the established range of typical adults, albeit at the upper end (>75^th^ percentile). Our hand transplant results suggest that these S1_a_ responses may change over time: each transplant patient had sessions in more than one of those four categories, and both T1 and T2 showed higher activation for their first sessions than their subsequent sessions. In summary, individual hand restoration patients showed no consistent pattern of S1_a_ responses to stimulation of the intact hand, though they never fell outside the established range of variability in typical adults.

During stimulation of the affected hand, hand restoration patients showed weak but extant S1_a_ responses, as shown in Figure 3B. All 8 sessions were “low activators” in that their responses fell below the 95% confidence interval of typical adults, and below the 30^th^ percentile of typical adults, a significantly higher rate than chance (Pearson's χ^2^ = 9.16, *p* = 0.025). While these S1_a_ responses were weak, they nevertheless represent a cortical response to stimulation of the restored hand. We found no patterns that distinguished hand transplants from hand replant patients. In summary, all hand restoration patients showed S1_a_ responses, albeit weak responses, to stimulation of the affected (i.e., restored) hand.

Our primary hypothesis did not cover S1 responses during stimulation of the face, because previous studies showed no deafferentation-related reorganization in response to face stimulation (Valyear et al., 2020). Nevertheless, we report these results to confirm that no unexpected reorganization occurred in hand restoration patients. Indeed, during stimulation of the face, most hand restoration sessions showed S1_a_ activity within the normal range of variability of both typical adults and amputees, as shown in Figures 3C,D. The one exception was replant participant R1 (blue points), who showed an elevated S1_a_ response to stimulation of the intact-side face.

Overall, hand restoration patients showed a wide variety of S1_a_ responses to tactile stimulation. During stimulation of the intact hand, S1_a_ responses (Figure 3A) were highly variable; patients were equally likely to respond like amputees as like typical adults (4/8 sessions were closer to amputee median, 4/8 to typical adult median), and all patient responses fell within the range of typical adults. Stimulation of the affected hand consistently led to grossly typical responses in contralateral S1_a_, though these responses were of low magnitude. Across stimulation sites, we found a few participant-specific patterns. First, replant patient SP (yellow points) was frequently a “low activator,” with the lowest or second-lowest activation magnitude during stimulation of intact hand or bilateral face. Transplant T1 (red points) consistently had the strongest magnitudes on their first session (T1s1), weaker on second (T1s2), and their third session (T1s3) was near their second. These patterns do not reveal any consistent differences between transplants and replants in S1_a_ response to tactile stimulation.

### Intact somatosensory cortex shows low-but-normal sensitivity to intact hand stimulation in hand restoration patients

In intact somatosensory cortex (S1_i_) of hand restoration patients, stimulation of the intact hand led to low-magnitude responses for most sessions, as shown in Figure 3E. Most participants (7/8 sessions, 88%) were “low activators,” with S1_i_ response magnitudes below the 30^th^ percentile of both comparisons groups; though with our small sample size, this rate was not significantly greater than chance (Pearson's χ^2^ = 5.95, *p* = 0.147). The one exception was replant WH (blue point), whose S1_i_ response was above the 75^th^ percentile of healthy adults. In summary, most hand restoration patients (88%) were “low activators,” but within the typical range, for S1_i_ responses to stimulation of the contralateral intact hand.

During stimulation of the affected hand, hand restoration patients showed typical S1_i_ responses, as shown in Figure 3F. 7/8 sessions had S1_i_ responses within the normal range of typical adults; the one exception was again replant R1 (blue point), whose response magnitude was greater than the typical range.

During stimulation of the face, most hand restoration sessions showed S1_i_ activity within the normal range of variability of both typical adults and amputees, as shown in Figures 3G,H. The two exceptions were transplant T1s1 (darkest red point), and replant R1 (blue point), who showed high-magnitude responses to bilateral face stimulation.

Overall, most hand restoration patients had S1_i_ responses within the typical range of responses to tactile stimuli. S1_i_ showed low-magnitude (but still within typical range) responses to stimulation of the intact hand. This effect did not directly involve affected systems (neither S1_a_ nor affected hand), suggesting widespread or secondary cortical plasticity specific to the hand restoration process. However, no such pattern arose for S1_i_ responses to stimulation elsewhere. Two participant-specific patterns arose clearly: first, transplant T1s1 was a “high activator” for bilateral face stimulation, though this effect disappeared for T1's later sessions. Second, replant R1 was a universal “high activator,” with S1_i_ responses above normal during stimulation at all sites.

### Patient sensation and demographics have no clear relationship with responses in somatosensory cortex

Our small group of hand restoration patients makes it difficult to quantify relationships between patient-specific factors and brain activity. Correlation statistics are not reliable at our sample size, but qualitative observation of our data (Supplementary Figure 1) shows no indication of any relationship between our four behavioral/demographic variables (locognosic error, years since amputation, years since surgery, age at test) and stimulation-based activity (% signal change).

### No evidence of global changes in cortical responsiveness for hand restoration patients

To test for possible changes in brain responses to non-tactile stimulation in non-sensorimotor areas following hand restoration (i.e., global changes in cortical responsiveness), we used a visual mapping task to identify brain areas responsive to viewing a flashing checkerboard stimulus. We found no differences in visual mapping responses between hand replant patients and our comparison groups, as described in Supplementary Figure 2.

### Hand restoration patients show reduced tactile localization, but it may improve over time

**Figure 4:**
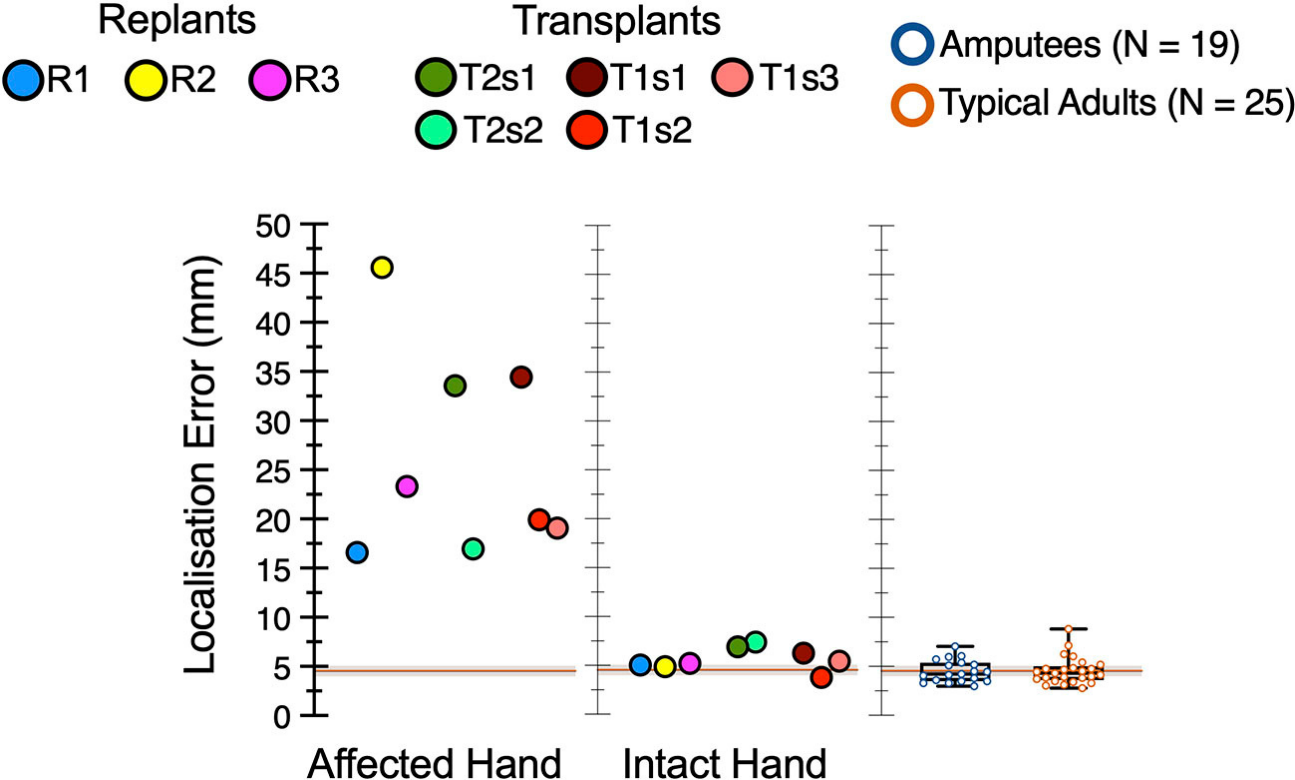
Elevated locognosic error in the restored hand. In the affected hand, all hand restoration patients' locognosic error was elevated compared to either comparison group (*p* < 1 x 10^−8^). For both transplant patients (T1 and T2), locognosic error was lower in later sessions compared to their first session.

All hand restoration patients showed elevated locognosic error (i.e., reduced tactile localization) on the affected hand, vs. both comparison groups (*t* > 8.61, *p* < 1 x 10^−8^), as shown in Figure 4.

In our hand transplant participants, tactile localization in the affected hand improved over time. Session (i.e., time) had a significant effect on locognosic error in the affected hand, both for patient T1 (*F*_(2, 91)_ = 3.89, *p* = 0.024; Figure 4 red points) and for patient T2 (*F*_(1, 86)_ = 15.25, *p* = 0.0002; Figure 4 green points). *Post-hoc* tests indicated that these effects arose because their first session had greater error than later sessions, in both participants. Note that the two inter-session gaps with improved sensation (T1s1-T1s2 = 455 days, T2s1-T2s2 = 385 days) were longer than the one without improved sensation (T1s2-T1s3 = 88 days), so changes in tactile localization may arise from the passage of time, but this is difficult to confirm from 3 samples across 2 participants.

No hand restoration patient showed referred sensation. As previously reported (Valyear et al., 2020), neither did any member of our amputee group.

## Discussion

We investigated cortical organization of the primary somatosensory complex (S1) in human hand restoration (transplant and replant) patients with well-controlled tactile stimulation, and compared these patients to typical adults and to amputees. We found that hand restoration patients largely showed S1 organization within the range of variability found in both typical adults and amputees, the fourth of our possible outcomes. Patients demonstrated substantial individual variability: during stimulation of the intact hand, the majority of patients' S1_a_ responses were more like amputees (i.e., within the 95% confidence intervals of the mean group estimate of responses from amputees, and above the 75^th^ percentile of typical adults), and some responses were like neither amputees nor typical adults (i.e., below the 25^th^ percentile of typical adults). During stimulation of the affected hand, all hand restoration patients showed S1_a_ responses within the range of typical variation, albeit of low magnitude. This confirms previous findings that, in hand restoration patients, S1_a_ has “grossly typical” organization (Frey et al., 2008), in that it responds to contralateral stimulation despite its lengthy deafferentation. However, our data reveal that the “grossly typical” nature of post-restoration organization may indeed be only *grossly* so: post-restoration S1_a_ activity may not match its original activity patterns. Despite the limitations of a case study approach, our findings describe S1 responses to well-controlled tactile stimulation in allogeneic hand transplant and autogenic hand replant patients. Because of the data's limits, we have presented the data simply with few minimal mathematical assumptions, to allow the reader to draw conclusions and to inform future research. This descriptive study uncovers numerous questions for further research in cortical organization for tactile sensation following deafferentation and re-afferentation.

### Hand restoration patients show s1a responses similar to typical adults and amputees, with substantial unexplained inter-individual variability

To our knowledge, the current study presents the only description of S1 responses to well-controlled tactile stimulation in hand transplant and replant patients. Overall, our hand restoration patients showed S1 responses to tactile stimulation within the normal range of variability found in typical adults. However, this range of variability is wide enough that it includes some patients whose S1_a_ responds to stimulation of the intact hand in a manner more like amputees than like typical adults.

All of our hand restoration patients showed S1_a_ responses to stimulation of the contralateral affected hand, which indicates grossly typical S1 organization, i.e., restored cortical responses to stimulation of the restored hand. This confirms previous findings from a case study of a single hand transplant patient with less well-controlled tactile stimuli (Frey et al., 2008), and is consistent with previous studies have found preserved movement- and possibly sensation-related information in sensory and motor cortex despite long-term disuse after amputation (Serruya et al., 2002; Hochberg et al., 2006; Makin et al., 2013b; Brandman et al., 2018; Flesher et al., 2021; Kikkert et al., 2021). Compared to the previous study of hand transplant patients, our larger samples of patients and comparison groups allowed us to identify that hand restoration patients universally showed low (below 30^th^ percentile) S1_a_ responsiveness to tactile stimulation, which indicates that “grossly typical” organization may not equal fully typical organization. This could arise from all patients' poor tactile sensory localization in the affected hand (Figure 4), but this explanation seems unlikely because most hand restoration patients showed low-end-of-typical responses to contralateral stimulation in *bilateral* S1: both in S1_a_ during stimulation of the affected hand (Figure 3B), and in S1_i_ during stimulation of the intact hand (Figure 3E). While we cannot rule out a role of peripheral factors (sensory localization, flawed reinnervation in hand restoration patients), they are unlikely to drive our findings given the absence of a relationship between peripheral sensory function (i.e., locognosic error) and responses in S1_a_ or S1_i_, as detailed in the next section.

Subcortical plasticity provides a second possible input-based alternative cause for apparent remapping of the cortex–in this case, from the cuneate nucleus, thalamus and/or spinal cord (Jones and Pons, 1998; Kambi et al., 2014; Halder et al., 2018). However, this concern applies equally to all studies of cortical reorganization, especially in humans where neuroimaging cannot easily assess plasticity in these subcortical sites.

Another explanation for low S1 activation in hand restoration patients is widespread secondary changes analogous to the bilateral cortical reorganization that occurs after unilateral deafferentation (Calford and Tweedale, 1990; Pelled et al., 2007, 2009; Pawela et al., 2010; Bogdanov et al., 2012; Valyear et al., 2020). Post-restoration bilateral changes would likely work through the same mechanisms that support interhemispheric transfer of plasticity after deafferentation, though post-restoration plasticity would likely reflect “re-reorganization,” which may not follow the specific patterns of deafferentation. While this provides a neurophysiological mechanism for bilateral changes in S1 responses after hand restoration, it is also possible that our low bilateral S1 responses reflect a sampling artifact, wherein our hand restoration patients happened to be individuals with low responses to contralateral hand stimulation. Sampling artifacts can never be ruled out in case studies.

Most of our hand restoration patients had an affected left hand, which introduces a potential confound in comparisons with our amputees, most of whom had an affected right hand. However, this is unlikely to be a major driver of our results, because our one patient with an affected right hand (transplant T2, green points) was not an outlier.

Our hand restoration patients showed substantial between-individuals variability, though the causes of this variability remain unknown and numerous. These patients' cases varied in mechanism of injury, time since surgery, rehabilitation history, and countless other characteristics. One goal of this study was to document the variability among hand restoration patients. We also found high within-individuals variability: both of our hand transplant patients had at least one session wherein their S1_a_ responded to intact hand stimulation more like amputees than typical adults (though still within the range of typical adult variability), and at least one session with typical-like S1_a_ responses. We found no systematic differences between hand transplant patients and hand replant patients, despite the substantial differences in the amount of time spent deafferented (transplant patients 12.3 and 2.6 years; replants ≤ 24 h), surgical constraints related to the emergency nature of replant surgery, and possible enhanced nerve regeneration in hand transplant patients due to immunosuppression (Mackinnon et al., 1987; Leonard et al., 1990). The similar S1 responses between the two groups suggests that the cortical consequences of hand restoration are robust to these peripheral differences and thus primarily driven by cortical processes.

### Hand transplant patients improve their sensory localization over time, but this is unaccompanied by changes in somatosensory cortex

Both of our hand transplant patients showed significant improvements in tactile sensory localization (i.e., lower locognosic error) between their first session and subsequent sessions. We saw no differences between patient T1's second and third session (T1s2 and T1s3), but this inter-session period (88 days) was smaller than the periods across which localization improved (T1s1-T1s2 455 days, T2s1-T2s2 385 days). However, despite these changes in localization, we found no clear pattern of accompanying changes in S1_a_ or S1_i_ responses to stimulation in the affected hand. For example, T1s1 showed an elevated S1_i_ response to left lower face stimulation, which disappeared by T1s2; however, we found no similar pattern between T2s1 and T2s2, and it is difficult to explain how a change in tactile localization in the hand could lead to altered responses to face *but not hand* stimulation.

The apparent dissociation between tactile localization and S1 response magnitude is not necessarily in conflict with our hypothesis in the previous section that low-magnitude S1 responses may be related to poor tactile localization at the group level, because all of our hand restoration patients had substantially elevated locognosic error compared to typical adults (Figure 4). For example, the relationship between S1 response magnitude and tactile localization could be categorical: some neural activity (e.g., integrative) may only occur if the inputs have the appropriate organization. Alternatively, S1 representations may be driven more by movement processes than by the passive sensation we tested here. A third possible explanation is that conscious awareness of sensation may depend more on higher-level sensory areas such as secondary somatosensory area S2 (Auksztulewicz et al., 2012; Preusser et al., 2015), or on distributed changes in the fronto-parietal network involved in conscious awareness of touch (Grund et al., 2021). However, these functional explanations remain purely theoretical. The low-magnitude S1 responses could arise from a broad rebalancing of cortical sensitivity, though the lack of altered sensitivity in the visual system suggests that the rebalancing is not a global cortical phenomenon. Instead, if the rebalancing is real, it is specific to sensory or sensorimotor cortex.

### Study limitations

It is possible that our data could be inadequate for the question at hand. For example, magnitude of S1 BOLD responses might not suffice to analyze the post-amputation (and post-restoration) responses to tactile stimuli. Tactile sensation > 3 years post-transplant should depend on cortical factors rather than peripheral factors, because peripheral nerve regrowth (~1 mm/day; Seddon et al., 1943) in the forearm and hand should be complete within 18 months. Peripheral factors such as axon growth and remyelination are *necessary* for restored function after nerve injury (Brushart, 2011), but not *sufficient*, as demonstrated by the 39% of upper extremity peripheral nerve injury patients who never achieve satisfactory recovery despite successful surgery (Dyck et al., 2005; He et al., 2014). Many studies have used BOLD magnitude to assess post-amputation cortical changes (e.g., Vargas et al., 2009; Sirigu et al., 2011; Philip and Frey, 2014; Kikkert et al., 2016), but if this approach cannot detect the reorganization associated with clear changes in function, future studies may benefit from the use of alternative measures such as representational similarity approaches (Walther et al., 2016; Berlot et al., 2019) to assess cortical mechanisms of tactile sensation.

Alternatively, our inability to detect a relationship between tactile localization and cortical reorganization could arise because task fMRI data have high inter-individual variability (e.g., Davis et al., 1998; Handwerker et al., 2004; Philip and Frey, 2014; Makin et al., 2015), which makes fMRI ill-suited to single-case analyses. Test-retest reliability for sensorimotor task fMRI is between good and excellent (Friedman et al., 2007), unlike cognitive task fMRI (Elliott et al., 2020), but even if the organization of somatosensory cortex contains stable and meaningful patterns, detection of these patterns may require dramatically more data than are collected in most studies. In resting state functional connectivity MRI, stable within-participant measurements require at least 500 images (Gordon et al., 2017); while this number is not directly comparable to a tactile stimulation study, it dramatically exceeds our ~154 images per stimulation site.

The current data also cannot adequately identify time-related effects. Without multiple sessions taken in close succession, we cannot distinguish true between-session effects from sampling artifacts or learning effects. Without pre-surgery data, we cannot establish true baselines. Such data are difficult to collect without extensive access to patients' time and lives, a challenging but worthwhile goal for studies of rare patient groups.

These limitations highlight the methodological challenges inherent to case studies. First, small sample sizes contain an unavoidable risk of sampling artifacts. Second, individual participants are difficult to classify when comparison group(s) have high between-individuals variability, as is frequently the case in fMRI. Third, analytic options may be limited depending on the nature of the data. Therefore, rare conditions deserve to be studied with a scientific approach that presents the data simply, with minimal mathematical assumptions, and allows the reader to draw conclusions. Toward this end, we have followed the same minimal statistical approach as previous case studies (Frey et al., 2008; Valyear et al., 2019). Overall, case studies remain valuable despite their methodological limits because rare patient groups (such as allogeneic hand transplant and autogenic hand replant patients) present unique situations that allow investigation of otherwise-inaccessible phenomena.

### Conclusions

We described S1 BOLD responses to tactile stimulation of the hands and lower face in 5 allogeneic hand transplant and autogenic hand replant patients. These individuals' S1_a_ responded to tactile stimulation within the range of typical adults, though S1_a_ responses to affected-hand stimulation were of generally low magnitude, and S1_a_ responses to intact hand-stimulation showed high between-individuals variability. Therefore, we found evidence that S1 may show a grossly typical pattern of activity after hand restoration, yet nonetheless we also found signs that this activity pattern may not be fully typical. At a group level, our results were inconclusive, but highlight the unknown spaces in our knowledge of how the brain's response to tactile sensation can change after de-afferentation and re-afferentation.

Tactile sensory localization on the affected hand was not correlated with magnitude of S1 response to stimulation. Moreover, hand transplant patients improved their tactile sensory localization over time, but these improvements were not accompanied by any consistent change in S1 BOLD responses to tactile stimulation of the affected hand. Together, these results suggest that S1 BOLD magnitudes may not capture the cortical plastic processes that underpin long-term improvements in tactile localization for these patients. Nevertheless, our well-controlled tactile stimulation allowed us to describe the variation between sensory processes and S1 organization in the rare neurological cases of allogeneic hand transplant and autogenic hand replant patients.

## Data availability statement

The raw data supporting the conclusions of this article will be made available by the authors, without undue reservation.

## Ethics statement

The studies involving human participants were reviewed and approved by University of Missouri Institutional Review Board. The patients/participants provided their written informed consent to participate in this study.

## Author contributions

BP: data curation, formal analysis, investigation, methodology, writing original draft, and writing review and editing. KV: formal analysis, investigation, methodology, visualization, and writing review and editing. CC and NB: investigation and methodology. CK: conceptualization and funding acquisition. SF: conceptualization, data curation, funding acquisition, methodology, project administration, resources, supervision, and writing review and editing. All authors approved the submitted version.

## Funding

This work was supported by National Institute of Neurological Disorders and Stroke at the National Institutes of Health [Grant # NS0833770], and the United States Department of Defense [Grant #W81XWH-13-1-0496] awarded to SF.

## Conflict of interest

The authors declare that the research was conducted in the absence of any commercial or financial relationships that could be construed as a potential conflict of interest.

## Publisher's note

All claims expressed in this article are solely those of the authors and do not necessarily represent those of their affiliated organizations, or those of the publisher, the editors and the reviewers. Any product that may be evaluated in this article, or claim that may be made by its manufacturer, is not guaranteed or endorsed by the publisher.
