# Corrigendum: Changes in primary somatosensory cortex following allogeneic hand transplantation or autogenic hand replantation

**DOI:** 10.3389/fnimg.2022.1127605

**Published:** 2023-01-12

**Authors:** Benjamin A. Philip, Kenneth F. Valyear, Carmen M. Cirstea, Nathan A. Baune, Christina Kaufman, Scott H. Frey

**Affiliations:** ^1^Department of Psychological Sciences, University of Missouri, Columbia, MO, United States; ^2^Program in Occupational Therapy, Washington University School of Medicine, St. Louis, MO, United States; ^3^School of Human and Behavioural Sciences, Bangor University, Bangor, United Kingdom; ^4^Department of Physical Medicine and Rehabilitation, University of Missouri, Columbia, MO, United States; ^5^Department of Cardiovascular and Thoracic Surgery, University of Louisville School of Medicine, Louisville, KY, United States

**Keywords:** amputation, somatosensory cortex, replantation, cortical organization, deafferentation, hand transplantation, tactile sensation

In the published article, there was an error in Table 1 as published. The participant identifiers were not consistent with the text and figures. The corrected Table 1 and its caption appear below.

**Table 1:** Demographics of hand restoration patients.

**Name**	**Aff. Side**	**Dominant Side**	**Type**	**Age**	**AAA**	**YSA**	**YSS**	**YD**
T1s1	L^*^	R	Transplant	38.6	23.1	15.5	3.2	12.3
T1s2	L^*^	R	Transplant	39.8	23.1	16.8	4.4	12.3
T1s3	L^*^	R	Transplant	40.1	23.1	17.0	4.7	12.3
T2s1	R	R	Transplant	49.2	41.1	8.1	5.5	2.6
T2s2	R	R	Transplant	50.3	41.1	9.1	6.6	2.6
R1	L	R	Replant	62.2	55.7	6.5	6.5	0
R2	L	R	Replant	59.8	59.5	0.3	0.3	0
R3	L	R	Replant	40.9	34.5	6.4	6.4	0

Aff., affected; L^*^, LH transplant, but RH also injured; AAA, age at amputation; YSA, years since amputation; YSS, years since transplant/replant surgery; YD, years deafferented (i.e., between amputation and surgery).

The original table is not shown in this corrigendum because the participant identifiers were changed to improve participant confidentiality. The only change is the values in the “Name” column.

The authors apologize for this error and state that this does not change the scientific conclusions of the article in any way. The original article has been updated.

