# Supplementary material for: Changes in primary somatosensory cortex following allogeneic hand transplantation or autogenic hand replantation
Source: Front Neuroimaging. 2022 Oct 6;1:919694. doi: 10.3389/fnimg.2022.919694 (PMC9802660; doi:10.3389/fnimg.2022.919694)
Supplement: Supplementary file 1 [file Table_1.DOCX]

Supplementary Material

# Supplementary Methods

## Visual Mapping Methods

Functional scan parameters for the visual mapping task differed as follows: TE = 4.38 ms, flip angle = 8.0 , FoV = 256 mm, and in-plane resolution at 3.125 by 3.125 mm. All other parameters were the same as those used for the somatosensory mapping task.

Visual mapping data ROI analysis was performed similarly to analysis of S1 ROIs, in order to quantify visual activation between groups. Visual ROIs were anatomically defined as all areas with ≥ 50% chance of being within any of the areas V1–V5, according to the Juelich Histological Atlas (Geyer et al., 2000) in FSL; separately for left and right hemisphere. Each group-defined ROI was inverse-registered with FLIRT to individual participant space, producing participant-specific ROIs. For statistical tests comparing hand replant visual mapping ROI data to the comparison groups, Crawford & Howell's Modified T-Test was used at α = 0.0125 (0.05 / 4) for four tests: S1_a_ and S1_i_ vs. typical adults and amputees.

# Supplementary Results

## Correlation Results: Patient Sensation and Demographics Do Not Predict Responses in Somatosensory Cortex

Our small group of hand restoration patients makes it difficult to identify relationships between patient-specific factors and brain activity. Despite the problematic nature of correlational tests at these sample sizes, we used correlation measures to illustrate possible relationships between our four behavioral/demographic variables (locognosic error, years since amputation, years since surgery, age at test) and stimulation-based activity (% signal change). We found no significant correlations under any analysis: not for either ROI, not for any stimulation site, not when combining groups (transplants and replants) nor when analyzing the groups separately. We found one trend that did not reach statistical significance after multiple-comparison correction: a trend toward a correlation between S1_i_ activity during intact hand stimulation and "years since amputation" (Kendall’s τ = -0.643, p = 0.03); all other pairings *p* > 0.10. Correlation statistics are not reliable at our sample size, so the relationships between behavioral/demographic variables and S1 activity are best observed directly in our data, shown in **Supplementary Figure 1**.

The main text and previous sections suggested no consistent differences between hand transplant vs replant patients, and we tested this statistically via Mann-Whitney U tests (nonparametric version of between-groups t-test). We found no instances with significant differences between hand transplant and replant patients. This was clear in most cases (p > 0.57), except for S1_i_ during intact hand stimulation, which showed a non-significant trend toward greater activation for replants (p = 0.07; **Supplementary** **Figure 1e**). Overall, surgery type – as with touch localization performance and demographics – did not predict S1 activity during tactile stimulation.

In case the above two analyses of sensation/demographics were distorted by the repeated-measures structure of our hand transplant patients, we repeated the above two analyses with each transplant reduced to a single across-sessions mean. As above, we found no evidence of correlation between behavioral/demographic variables and S1 activity (|τ| ≤ 0.8, *p* > 0.08), and no evidence of an effect of surgery type (*p* = 0.80).

## Visual Mapping Results: No differences in visual cortex between hand replants and comparison groups, but individual hand replant patients show consistent patterns

To test for possible changes in brain responses to non-tactile stimulation in non-sensorimotor areas following hand restoration (data available for replant patients only), we used a visual mapping task to identify brain areas responsive to viewing a flashing checkerboard stimulus. At a between-groups level, whole-brain analysis revealed no areas showing significant differences between hand replant patients and either comparison group, and Kruskal-Wallis tests (Kruskal and Wallis, 1952) on visual ROI data (see Methods) revealed no significant main effects of Group, neither for the left hemisphere (χ^2^ (2,46) = 4.44, p = 0.108) nor right hemisphere (χ^2^ (2,46) = 4.30, p = 0.117).

To test for possible relationships between individual participants’ brain responsiveness to visual and tactile stimulation, we evaluated correlations between visual cortex response to visual stimulation and S1 response to tactile stimulation. Statistical results are difficult to assess because of the small sample size (visual mapping data only available for replant participants, n = 3). However, comparing S1 vs. visual cortex responses in individual replants (**Supplementary Figure 2**) reveals that our S1 high activator (WH, blue) also showed outlying activation levels in visual cortex as a “low activator.” However, after multiple comparison correction, WH’s visual mapping response was not significantly different from the visual mapping response of the typical adult group (S1_a_ p = 0.019, t = -2.20; S1_i_ p = 0.027, t = -2.03) or the amputee group (S1_a_ p = 0.067, t = -1.65; S1_i_ p = 0.110, t = -1.32).

Overall, we found no differences in visual mapping responses between hand replant patients and our comparison groups. Our individual hand replant patient (WH) who frequently had strong S1 responses to tactile stimulation also trended toward distinctly weak visual cortex responses to visual stimulation.

# Supplementary Figures

**
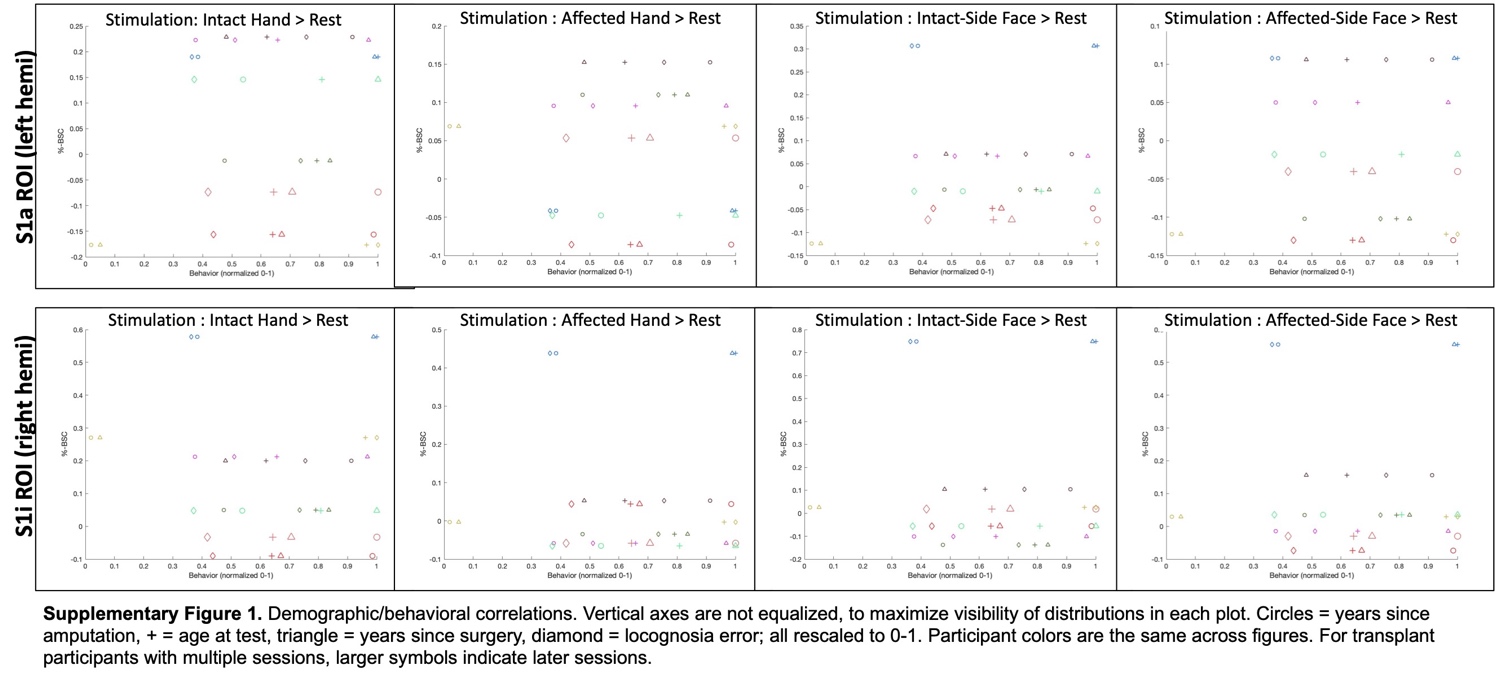
**

**Supplementary Figure 1.** Demographic/behavioral correlations. Vertical axes are not equalized, to maximize visibility of distributions in each plot. Circles = years since amputation, + = age at test, triangle = years since surgery, diamond = locognosic error; all rescaled to 0-1. Participant colors are the same across figures. For transplant patients with multiple sessions, larger symbols indicate later sessions.


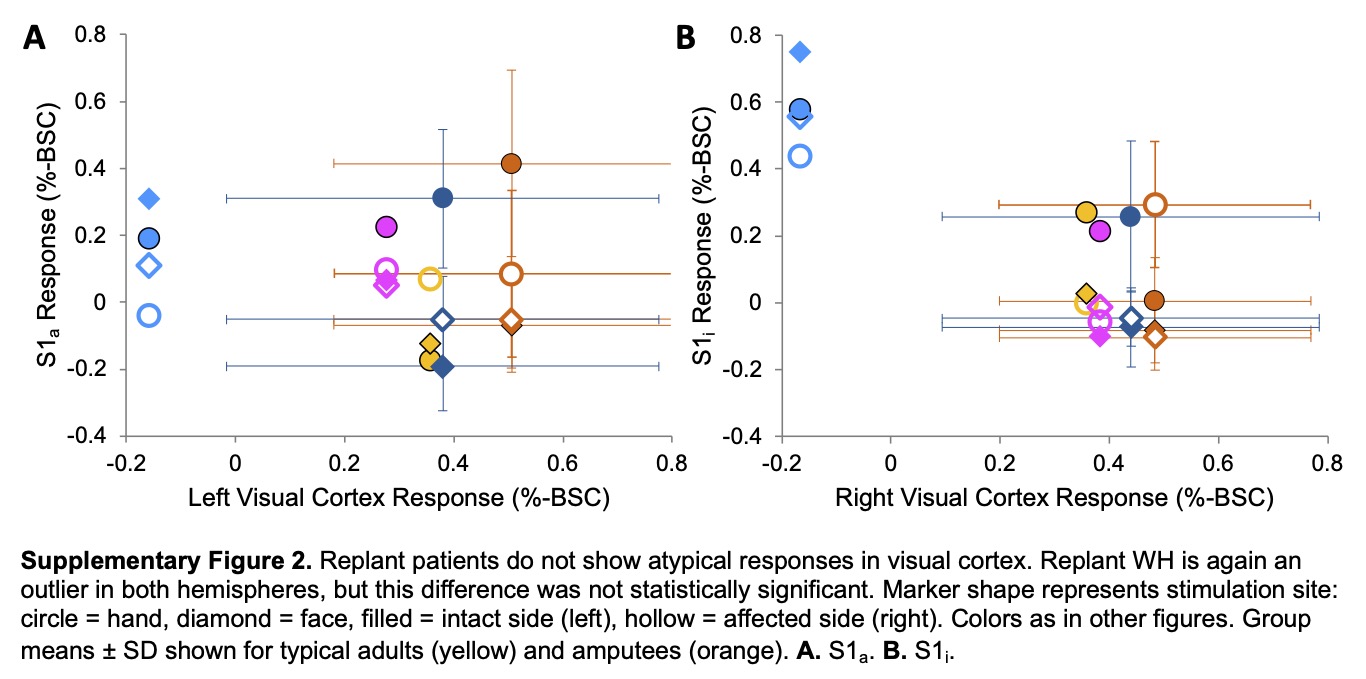


**Supplementary Figure 2.** Replant patients do not show atypical responses in visual cortex. Replant WH is again an outlier in both hemispheres, but this difference was not statistically significant. Marker shape represents stimulation site: circle = hand, diamond = face, filled = intact side (left), hollow = affected side (right). Participant colors are the same across figures. Groups means ± SD shown for typical adults (yellow) and amputees (orange). **A.** S1_a_. **B.** S1_i_.
